# Medical Mechatronics for Healthcare

**DOI:** 10.1155/2018/9675482

**Published:** 2018-04-18

**Authors:** Yi-Hung Liu, David Moratal, Javier Escudero, Han-Pang Huang

**Affiliations:** ^1^Department of Mechanical Engineering, National Taipei University of Technology, Taipei, Taiwan; ^2^Department of Electronics Engineering, Universitat Politècnica de València, Valencia, Spain; ^3^School of Engineering, Institute for Digital Communications, University of Edinburgh, Edinburgh, UK; ^4^Department of Mechanical Engineering, National Taiwan University, Taipei, Taiwan

## 1. Introduction

Advances in the healthcare technology have positioned biomedical technology as a major driver in global knowledge-based economies. A successful healthcare intervention depends on not only the capability or experience of clinicians but also the adequacy of medical instruments and assistive devices. In addition, the technical aids and assistive devices for elderly or people with severe motor disability are getting more attention due to our aging society all over the world, and they are widely used in daily life. As a result, medical mechatronics becomes an important emerging technology to improve healthcare. Medical mechatronics is the integration of technologies and knowledge from various domains [1], including biosignal sensing fusion, real-time clinical data analysis, electric and mechanical system design, assistive/rehabilitation robot development, and machine/deep learning algorithms. Although medical mechatronics has proven to be successful in healthcare applications, there still remain difficulties and challenges to overcome. For example, most previous assistive devices/robots were developed to provide patients with rehabilitation training in hospitals. With the rapid growth of aging population, these assistive devices are required to have smaller size and cheaper production cost and be safer in order to meet the requirement of in-house rehabilitation [2]. As a result, the medical mechatronic components in these assistive devices/robots need to be redesigned.

The goal of this special issue is to bring together the researchers in these fields and present high-quality research on recent developments on medical mechatronics and novel applications of medical mechatronics in healthcare, as well as the relevant prospect on opportunities and challenges. The selected eleven papers underwent a rigorous refereeing and revision process. Most of the studies were carried out on clinical data, which provides the results reported in this special issue a high confidence level. Moreover, most of the papers in this special issue include mechatronics and healthcare experts as coauthors, which is beneficial to open new methods and issues to medical experts in this multidisciplinary field.

## 2. The Special Issue

Stroke is a leading cause of long-term disability, and virtual reality- (VR-) based stroke rehabilitation is effective in increasing motivation and the functional performance in people with stroke. Although much of the functional reach and grasp capabilities of the upper extremities is regained, the pinch movement remains impaired following stroke. In the study by S.-C. Yeh et al., a haptic-enhanced VR system is proposed to simulate haptic pinch tasks in order to assist in long-term poststroke recovery of upper extremity fine motor function. Their results also suggest that this system is also effective under certain challenging conditions such as being in the chronic stroke phase or a coside of lesion and dominant hand (nondominant hand impaired).

In addition to VR-assisted rehabilitation, rehabilitation/mobile robot plays also a critical role in healthcare. This special issue collects a set of papers involving rehabilitation/healthcare robots. Ankle rehabilitation exercises act an important role in recovering walking ability of patients after stroke. Currently, patients mainly perform ankle exercise to reobtain range of motion (ROM) and strength of ankle joint under therapist assistance by manual operation. However, most of the rehabilitation devices focus on ankle functional training and ignore the importance of neurological rehabilitation in the early hemiplegic stage. Q. Liu et al. developed a novel robotic ankle rehabilitation platform to assist patients in executing ankle exercise. This robotic platform consists of two three-DOF symmetric layer-stacking mechanisms, which can execute ankle internal/external rotation, dorsiflexion/plantarflexion, and inversion/eversion exercises while the rotation center of the distal end of the robotic platform always coincides with patient's ankle pivot center. Y. Feng et al. proposed a new applicable and effective sitting/lying lower limb rehabilitation robot (LLR-Ro), which has a mechanical limit protection, an electrical limit protection, and a software protection to prevent the patient from being secondary damaged. As a new type of the rehabilitation robots, its hip joint rotation ranges are different in the patient sitting training posture and lying training posture. The mechanical leg of the robot has a variable workspace to work in both training postures. In addition, to eliminate accident interaction force between patients and LLR-Ro in the process of the passive training, an amendment impedance control strategy based on position control is also proposed to improve the compliance of the LLR-Ro. On the other hand, mobile robotics is a potential solution to home behavior monitoring for the elderly. For a mobile robot, there are several types of uncertainties for its perceptions, such as the ambiguity between a target object and the surrounding objects. The problem could be more serious for a home behavior monitoring system, which aims to accurately recognize the activity of a target person, in spite of these uncertainties. W. Yu et al. proposed a new strategy of active sensing, called active sensing with categorized further explorations. It detects irregularities and categorizes situations requiring further explorations, which strategically maximizes the information needed for activity recognition while minimizing the costs. Two schemes of active sensing, based on two irregularity detections, namely, heuristic-based and template-matching-based irregularity detection, were implemented and examined for body contour-based activity recognition. Their proposed approach can guide the robot system to sense the target person actively and achieve high accuracy of activity recognition.

Biceps brachii muscle illness is one of the common physical disabilities that requires rehabilitation exercises in order to build up the strength of the biceps brachii muscle after surgery. It is also important to monitor the condition of that muscle during the treatment or rehabilitation exercise. Electromyography (EMG) is one of the preferred methods for measuring and recording the activity of the biceps brachii muscle, and wavelet transform (WT) has been widely used as a temporal-spectral analysis method for monitoring EMG signals. However, WT parameter selection remains a challenging task. N. Burhan et al. analyzed and investigated the selection of the best mother wavelet (MWT) function and depth of the decomposition level in the wavelet denoising EMG signals of the biceps brachii muscle. The efficacy of the wavelet denoising signal was determined through an analysis of the activity of the biceps brachii muscle for vicenarians during the rehabilitation exercise.

Overnight polysomnography (PSG) is a standard diagnostic procedure for obstructive sleep apnea (OSA). However, there are no sensor systems to hook up with PSG for accurate head position monitoring available clinically. W.-Y. Lin et al. presented a CORDIC- (COordinate Rotation DIgital Computer-) based tilting sensing algorithm to quickly and accurately convert accelerometer raw data into the desired head position tilting angles. Their system can hook up with PSG devices for diagnosis to have head position information integrated with other PSG-monitored signals. It has been applied in an IRB test in Taipei Veterans General Hospital and been proved that it can meet the medical needs of accurate head position monitoring for PSG diagnosis. In addition to the head-posture monitoring during sleep, vibration condition monitoring is also a crucial factor in sleep study. H. Kimura et al. developed a new mechanical bed for inducing sleep to investigate the effects of different vibration conditions. The new bed has two active DOFs, vertical and horizontal directions, to examine the anisotropy of sensation. The bed includes three main parts: a vertical driver unit, horizontal driver unit, and unique 2-DOF counterweight system. Due to the new counterweight system, the required torque is extremely small and the driving sound is suppressed to less than 40 dB. Their results have suggested the ability of appropriate vibration to induce sleep.

C.-H. Kuo et al. proposed an oscillometric blood pressure (BP) measurement approach based on the active control schemes of cuff pressure. Compared with conventional electronic BP instruments, their proposed BP measurement approach is based on the utilization of a variable-volume chamber which can better actively and stably alter the cuff pressure during inflating or deflating cycles, because the variable-volume chamber could significantly eliminate the air turbulence disturbance during the air injection stage when compared to an air pump mechanism. C.-Y. Lin and P.-J. Hsieh developed an automatic dispensing system for Chinese herbal decoctions with the aim of reducing manpower costs and the risk of mistakes. They employed machine vision in conjunction with a robot manipulator to facilitate the grasping of ingredients. An offline least square curve fitting method was used to calculate the amount of material grasped by the claws and thereby improve system efficiency as well as the accuracy of individual dosages. Their experiments on the dispensing of actual ingredients have demonstrated the feasibility of their proposed system.

In the study of A. Chromy and O. Klima, a 3D scan model and thermal image data fusion algorithms are presented. At present, medical thermal imaging is still considered a mere qualitative tool enabling us to distinguish between but lacking the ability to quantify the physiological and nonphysiological states of the body. Such a capability would, however, facilitate solving the problem of medical quantification. Accordingly, they proposed a generally applicable method to enhance captured 3D spatial data carrying temperature-related information. Their method can be utilized for high-density point clouds or detailed meshes at a high resolution but is conveniently usable in large objects with sparse points. Also, the technique offers a wide application potential in medicine and multiple technological domains, including electrical and mechanical engineering.
